# Relevance of Spike/Estrogen Receptor-α interaction for endothelial-based coagulopathy induced by SARS-CoV-2

**DOI:** 10.1038/s41392-023-01488-3

**Published:** 2023-05-19

**Authors:** Silvia Stella Barbieri, Franca Cattani, Leonardo Sandrini, Magda Maria Grillo, Alessandra Amendola, Carmen Valente, Carmine Talarico, Daniela Iaconis, Gabriele Turacchio, Miriam Lucariello, Lucia Lione, Erika Salvatori, Patrizia Amadio, Gloria Garoffolo, Mariano Maffei, Francesca Galli, Andrea Rosario Beccari, Giuseppe Sberna, Emanuele Marra, Marica Zoppi, Michael Michaelides, Giuseppe Roscilli, Luigi Aurisicchio, Riccardo Bertini, Marcello Allegretti, Maurizio Pesce

**Affiliations:** 1 Centro Cardiologico Monzino, IRCCS, Milano, Italy; 2 Dompé farmaceutici S.p.A., L’Aquila, Italy; 3 Istituto Nazionale per le Malattie Infettive Lazzaro Spallanzani, Roma, Italy; 4 Institute of Experimental Endocrinology and Oncology “G. Salvatore” (IEOS), National Research Council, Naples, Italy; 5 EXSCALATE, Dompé Farmaceutici S.p.A, Napoli, Italy; 6 Takis S.r.l., Rome, Italy; 7 National Institute on Drug Abuse Intramural Research Program, Baltimore, 21224 MD USA; 8 Johns Hopkins School of Medicine, Baltimore, MD USA; 9 Atreius S.a.s., L’Aquila, Italy

**Keywords:** Molecular medicine, Computational biology and bioinformatics

**Dear Editor**,

The severe coagulation syndrome in numerous organs is the major life-threatening conditions characterizing the acute infection by SARS-CoV-2. Endothelial inflammation/dysfunction, platelet hyper-reactivity, generation of neutrophil extracellular traps, promote the activation of the coagulation cascade in an infection-dependent manner.^1^ The outcome of the COVID-19 vaccination campaign has also revealed a potential risk of vaccine-induced immune thrombotic thrombocytopenia (VITT), or thrombosis with thrombocytopenia syndrome (TTS).^1^ Although this risk is extremely low, the propensity of the Spike protein to induce inflammatory and coagulation factors in placental and endothelial cells (ECs)^2,3^ has called for caution during the SARS-CoV-2 vaccination campaign.

We recently described a potential function of Spike (S) as a co-factor for estrogen receptor alpha (ERα) nuclear signaling,^4^ mediated by interaction of a nuclear receptor co-regulator (NRC) LXD-like motif present on the viral protein S2 subunit, with the activation function 2 (AF-2) region on ERα. To clarify the relevance of this interaction for the expression/release of pro-coagulation factors by human ECs, we exposed the immortalized Ea.Hy926 EC line to increasing concentrations of wild-type (wt) S, 17β-Estradiol (ES; a natural ERα agonist) Tumor Necrosis Factor-alpha (TNFα; a pro-inflammatory cytokine involved in SARS-CoV-2 coagulopathy), Raloxifene and Fulvestrant (RAL, FS; two ERα inhibitors with a function as Selective Estrogen Receptor Modulator, SERM, and Degrader SERD, respectively). Incremental amounts of the S-protein increased the pro-coagulant activity (PCA) of the cells (Supplementary Fig. 1a, c) and the expression of Tissue factor (*TF*) mRNA (Supplementary Fig. 1b, d). The ability of the two ERα inhibitors to reduce cellular *TF* mRNA and viral secretion (measured by RT-PCR amplification of the *ORF1ab* transcript in the supernatant) was confirmed in real infection experiments performed on Ea.Hy926 cells with SARS-CoV-2 (Fig. 1a, b, Supplmentary Fig. S2).

**Fig. 1:**
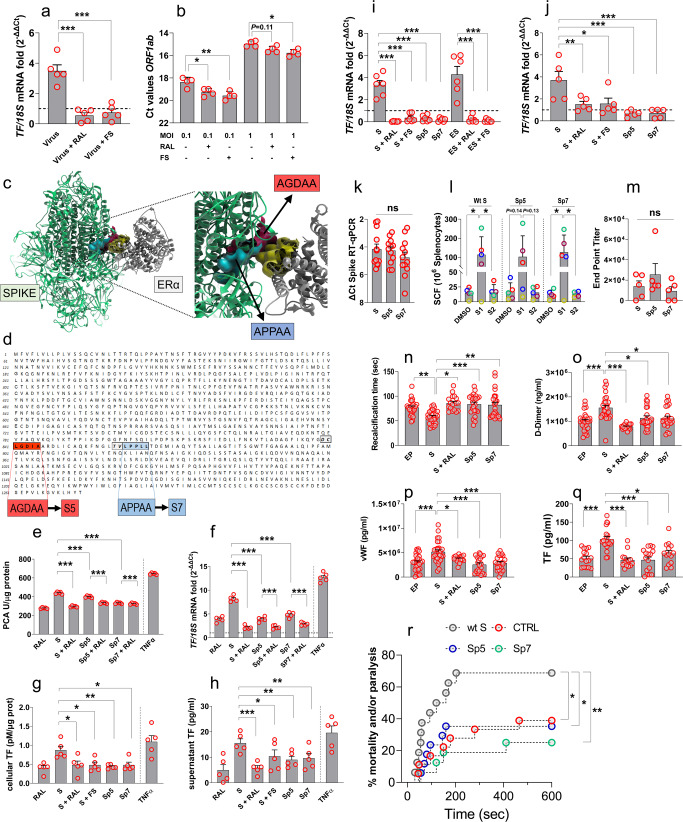
Effect of SARS-CoV-2 Spike and its Sp5/Sp7 mutants on EC coagulation cascade. **a** Infection of Ea.Hy926 endothelial cell line (1MOI) with SARS-CoV-2 enhances expression of *Tissue Factor* (*TF*) mRNA. The addition of ERα selective inhibitors Raloxifene (RAL) and Fulvestrant (FS) reduces the TF overexpression to the level of non-infected cells (equalized to 1 and represented with a dotted line in the bar graph). Data are represented as fold changes RNA expression in infected vs. non-infected cells. **b** Addition of ERα selective inhibitors also reduces the expression of the viral *ORF1ab* after infection with SARS-CoV-2 at 1 and at even 0.1 MOI, suggesting inhibition of viral replication in ECs. Data are expressed as Ct values. **c** Computer-modeled S-protein/ERα proteins structures and interaction. The region (helix-12) of the ERα interacting with S-protein is highlighted in yellow, while the two LDX motifs in S-protein are indicated as red and light blue surfaces, respectively. Sp5 and Sp7 mutations are indicated in the red and the light blue surface, respectively. **d** Primary aminoacidic sequence of the S-protein with an indication of the two LXD-like patterns evidenced in the red and light blue boxes, respectively. −1 and −2 positions are reported in italic and light gray background. **e**–**h** Pro-coagulant activity (PCA), *TF* mRNA, TF activity, and TF released in the culture supernatant of ECs exposed to wt S-protein, Sp5, or Sp7 mutants with or without Raloxifene (RAL) or Fulvestrant (FS). **i** Validation of wt S (±ERα inhibitors), Sp5 and Sp7 effect on primary human lung endothelial cells (HULEC). As shown by the fold increase in treated vs. untreated cells (equalized to 1 and represented with a dotted line in the bar graph), exposure to wt S, but not to Sp5 or Sp7 mutants determined upregulation of *TF* mRNA. This effect was bunted by RAL and FS. Estradiol (ES) ± ERα inhibitors were used as positive controls. **j** Pseudo-viruses engineered with wt S, Sp5, and Sp7 mutants were used in infection experiments (0.3 MOI) to validate the effect of S/ERα interaction on expression of *TF* mRNA. As shown, the pseudo-virus containing the wt S increased significantly expression of the coagulation factor compared to untreated cells (equalized to 1 and represented with a dotted line in the bar graph), or to the two pseudo-viruses bearing the S mutants or the combined treatment of wt S with ERα inhibitors. **k** Expression of the Spike transcripts (wt, Sp5, and Sp7 variants) at 48 h after electroporation of the expression vector in the adductor muscles in mice. Data are expressed as ΔCt values. **l**, **m** Cell-mediated and antibody-mediated immunity in mice electroporated with vectors bearing the wt S and Sp5/Sp7 variants. **n**–**q** In vivo coagulation response (expressed as a recalcification time) of plasma collected at 48 h after inoculation with vectors carrying the wt S-protein protein and Sp5/Sp7 mutants, and plasmatic levels of crucial coagulation markers in COVID-19 (D-Dimer, von Willebrand factor, TF). **r** In vivo thromboembolic effect of wt S, Sp5, and Sp7. The administration of the expression vector carrying the wt S increased the thrombosis events (evaluated as time for paralysis and/or death), as represented by the different trend represented in the graph. Significance of the difference between the curves of the treatments vs. wt Spike was assessed by Mantel-Cox Log Rank test. In vitro data (**a**, **b**, **e**–**j**) were analyzed with one-way ANOVA (repeated measures) with Tukey post hoc comparisons, while results of in vivo tests (**g**, **l**–**q**), were analyzed with unpaired one-way ANOVA with Tukey or Newman-Keuls multiple comparisons post-hoc tests. When tests did not reach statistical significance, the exact *P* value is indicated above each comparison, or indicated as ns. In every graph, the number of independent experimental replicates and animals is indicated by the number of the circles overlapped to graphs. The different colors of the dots in panel **l** represent the difference in the cell-mediated immune response of splenocyte from each mouse with respect to the challenging conditions (DMSO vs. S1 or S2 region) or the electroporation with the S-protein or the two Sp5/Sp7 mutant versions. In all graphs *P < 0.05, **P < 0.01 and ***P < 0.0001

The knowledge based on the EXSCALATE supercomputing platform^4^ guided us in introducing point mutations in the putative LXD-like motifs present in the S2 domain of Spike mimicking Nuclear Receptor Coactivator (NCOA) function. These regions—not related to known amino acid mutations reported in SARS-CoV-2 variants (Fig. 1c, d), were mutated into sequences (Sp5 and Sp7) with a potentially lower affinity for ERα, but maintaining a wild-type conformation. We first assessed the effect of the trimeric wt-S, Sp5, and Sp7 on the PCA and *TF* expression in ECs. The results showed a significant reduction of the PCA, and a decreased *TF* transcript and activation levels (Fig. 1e–h). Interestingly, *TF* expression and activity after treating cells with the two mutants were similar to the levels observed in RAL and FS treatments, thus validating the selectivity of the wt-S intracellular function *via* the interaction with ERα. These results were also validated in a line derived from human lung ECs (HULEC-5a), confirming the clinical relevance of our findings (Fig. 1i).

To exclude that interaction of other viral proteins with ERα accounts for the wt-S-induced *TF* upregulation, we generated a recombinant Vesicular Stomatitis Virus, in which the viral envelope glycoprotein was replaced by the wt-S or the Sp5/Sp7 mutants. Characterization of these pseudo-viruses (PSVs) by electron microscopy and preliminary infection in Ea.Hy926 cells indicated a similar content of the three variants (Supplementary Fig. 3), while *TF* mRNA was clearly expressed at lower levels in cells infected with PSVs containing the mutated Spike variants compared to wt-S (Fig. 1j). The wt-S and the Sp5/Sp7 coding sequences were also introduced into an expression vector designed to elicit immune responses similarly to expression systems employed in SARS-CoV-2 vaccination.^5^ Vectors were amplified and electroporated in the lower limb adductor muscles in mice. As shown in Fig. 1k, the three Spike variants were expressed at comparable levels, and this was accompanied by a comparable immunization of the mice against the S1 domain of the protein as assessed by ELISpot performed with splenocytes derived from the mice receiving the wt and the Sp5/Sp7 variants (Fig. 1l). Immunization was also confirmed by detection of anti-Spike antibodies in the plasma of the injected mice (Fig. 1m). To verify the coagulation response in vivo, the recalcification time as well as the levels of D-Dimer, von Willebrand Factor (vWF) and TF were measured in the plasma of control, wt-S, Sp5, and Sp7 electroporated mice. Results showed a transient increase in blood clotting at 48 h followed by a return to basal levels at 96 h (Fig. 1n, S4) in mice treated with wt-S, but not Sp5 and Sp7. The analysis of circulating coagulation markers characterizing the COVID-19 infection^6^ produced similar results (Fig. 1o–q). These data were finally validated by employing a disseminated thrombosis in vivo model, where thromboembolic death/paralysis after collagen/epinephrine injection was much lower in Sp5/Sp7-treated than in wt-S-treated mice (Fig. 1r).

Although vasculopathy consequent to COVID-19 depends on the damage/inflammation of the endothelium mediated principally by ACE-2, other mechanisms are emerging even not directly related to the infective activity of the virus. For example, sensing of the E protein by inflammatory cells causes a strong innate immunity reaction mediated by interaction with TLR2,^7^ and vasculopathy in the heart could be determined by disruption of the vascular homeostatic function of pericytes due to the activation of CD147 receptor signaling by the Spike S1 domain.^8^ By providing evidences that the activation of coagulative cascade might derive from the interaction of Spike with ERα, the present study establishes, for the first time, an important function of the viral protein as a co-factor for induction of TF, and of other coagulation effectors (D-Dimer, vWF) in COVID-19.^6^ Various mechanisms could account for this elevation according to various possibilities. For example, it is possible that after internalization of the viral protein, the Spike/ER complex translocates into the nucleus to directly activate transcription of ER-dependent targets. A second possibility is that the pro-coagulation effects of Spike occur mostly *via* the membrane-bound ER, and thus *via* a second-messenger mechanism. While this extranuclear function of Spike on activation of the coagulation cascade via ERα could be reconciled with what found recently on the release of vWF by endothelial cells, mediated by an ACE-2 independent interaction with the cytoskeleton-associated protein-4,^9^ it does not explain the *TF* transcriptional upregulation observed in our experiments.

In summary, also corroborated by mounting evidences from clinical and in vitro studies showing the potential effectiveness of SERMs (e.g., Raloxifene) as an anti-viral agent in COVID-19,^10^ our results suggest a new non-infective pathologic action of the S-protein at the vascular level enhancing the endothelial pro-coagulation activity. Given the residual risk of coagulopathy observed in subjects treated with COVID-19 vaccines, our study indicates two variants of the original Spike sequence that could be employed to design new versions of COVID-19 vaccines lacking any residual risk of VITT in the still ongoing vaccination and boosting campaign.

## Supplementary information

supplementary information

## Data Availability

The raw data introduced in the present manuscript are available upon reasonable request to the corresponding Authors.
