# Supplementary material for: Relevance of Spike/Estrogen Receptor-α interaction for endothelial-based coagulopathy induced by SARS-CoV-2
Source: Signal Transduct Target Ther. 2023 May 19;8:203. doi: 10.1038/s41392-023-01488-3 (PMC10196308; doi:10.1038/s41392-023-01488-3)
Supplement: Supplementary file 1 — supplementary information [file 41392_2023_1488_MOESM1_ESM.docx]

**Relevance of Spike/Estrogen Receptor-α interaction for endothelial-based coagulopathy induced by SARS-CoV-2**

Silvia Stella Barbieri^1, #^, Franca Cattani^2, #^, Leonardo Sandrini^1^, Magda Maria Grillo^1^, Alessandra Amendola^3^, Carmen Valente^4^, Carmine Talarico^5^, Daniela Iaconis^4^, Gabriele Turacchio^4^, Miriam Lucariello^4^, Lucia Lione^6^, Erika Salvatori^6^, Patrizia Amadio^1^, Gloria Garoffolo^1^, Mariano Maffei^2^, Francesca Galli^2^, Andrea Rosario Beccari^3^, Giuseppe Sberna^3^, Emanuele Marra^5^, Marica Zoppi^1^, Michael Michaelides^7,8^, Giuseppe Roscilli^6^, Luigi Aurisicchio^6^, Riccardo Bertini^9^, Marcello Allegretti^2, *^ and Maurizio Pesce^1, *^

**Supplementary information**

***3D Proteins Selection and molecular dynamics simulation workflow***

The S-protein 3D model was built based on the experimental coordinates deposited on the Protein Data Bank database with code 6VYB returned to its wild-type form and fully glycosylated [^1^](#_ENREF_1)*.* For the estrogen receptor, the X-RAY PDB model with code 3UUD was used, containing ERα and Nuclear receptor coactivator-2 [^2^](#_ENREF_2) and 3OLL, containing ERβ and Nuclear receptor coactivator-1[^3^](#_ENREF_3)*.* The S-protein 3D model was built based on the experimental coordinates deposited on the Protein Data Bank database with code 6VYB returned to its wild-type form and fully glycosylated [^1^](#_ENREF_1)*.* An asymmetric glycosylation of the three protomers has been derived by glycoanalyitic data for the N-glycans and O-glycans as previously reported [^3^](#_ENREF_3). For the estrogen receptor, the X-RAY PDB model with code 3UUD was used, containing ERα and Nuclear receptor coactivator-2 [^2^](#_ENREF_2) and 3OLL, containing ERβ and Nuclear receptor coactivator-1[^3^](#_ENREF_3)*.* The proteins were modeled using Amber14SB force field [^4^](#_ENREF_4), the carbohydrate moieties by the GLYCAM06j-1 version of GLYCAM06 force field [^5^](#_ENREF_5), and the general amber force field (GAFF) was used for the estradiol bound to ER receptor. The so prepared structure was used as starting point for MD simulations. Protein was inserted in a clinic box, extending up to 10 Å from the solute, and immersed in TIP3P water molecules. Counter ions were added to neutralize the overall charge with the genion GROMACS tool. After energy minimizations, the system was relaxed for 5 ns by applying positional restraints of 1000 kJ mol^−1^ nm^-2^ to the protein atoms. Following this step, unrestrained MD simulation was carried out with a time step of 2 fs, using GROMACS 2020.2 simulation package (supercomputer Marconi-100, CINECA, Bologna, Italy) V-rescale temperature coupling was employed to keep the temperature constant at 300 K. The Particle-Mesh Ewald method was used for the treatment of the long-range electrostatic interactions. The first 5ns of each trajectory were excluded from the analysis. The trajectory obtained after 1 microsecond MD simulation has been clustered in order to obtain representative structures. In particular, the structure used for the docking studies is the first centroid of the first cluster extracted from the MD experiment. For the ER, the XRAY PDB model with code 3OLL was used, containing 17β-Estradiol and Nuclear receptor coactivator 1[^6^](#_ENREF_6).

***S-ERα Protein-Protein Docking procedure***

The input of two individual proteins were set up. In particular, the S-protein and ER were used as receptor and ligand respectively. Then, the HDOCK tool was run to sample putative binding modes through an FFT-based search method and then scoring the protein–protein interactions. Then, the top 100 predicted complex structures are produced as output, and the best ten hypotheses were visually inspected to confirm the reliability of the calculation. The entire workflow is well described in the work published by Yan et al [^7^](#_ENREF_7).

***Manufacturing of S-protein, Sp5 and Sp7 for in vitro testing***

The nucleotide sequences encoding for the trimeric wt-S, Sp5 and Sp7 S-proteins and the different mutants were generated and inserted into a plasmid DNA vector for manufacturing as recombinant proteins in HEK293 cells. This vector encompassed the Human cytomegalovirus (CMV) immediate early enhancer and promoter, the murine Ig kappa chain leader sequence for protein secretion, the bovine growth hormone polyadenylation (bGH-PolyA) signal, a specialized termination sequence for protein expression in eukaryotic cells and the kanamycin resistance gene from *Staphylococcus aureus* for plasmid amplification in bacteria. The trimeric S-protein and its mutants were produced by transient transfection of Expi293F high-density cells with the ExpiFectamine 293 (Thermo Fisher) lipid cationic transfection reagent according to the manufacturer's instructions. The supernatant containing the proteins was collected after five days of incubation from start of transfection and subjected to clarification by centrifugation and filtration for the subsequent purification steps. The proteins were batch purified using IMAC (Immobilized Metal Chelate Affinity Chromatography) using PureCube Ni-NTA Agarose resin slurry (Cube-Biotech). Briefly, the resin slurry was centrifuged and incubated with equilibration buffer (50mM NaH_2_PO_4_, 500mM NaCl, pH 7.4, 10mM imidazole). The equilibrated resin was combined with the culture supernatant containing the recombinant proteins and incubated o/n, 4°C, on a rotating platform. The resin was subsequently collected by centrifugation, washed, and the protein was eluted by an elution buffer containing 300 mM Imidazole and subjected to dialysis in phosphate buffer (PBS) using slide-A-lyzers (Thermo Fisher) as indicated in the product datasheet. Once recovered from dialysis, the proteins were quantified on a spectrophotometer measuring the absorbance at 280 nm. Trehalose (Sigma-Aldrich) was used (5% w/v) as a stabilizer of *in vitro* produced S-proteins[^8^](#_ENREF_8). The purity of the proteins was assessed by SDS-PAGE and Western Blot analysis, conducted under both reducing and non-reducing conditions. The obtained KD (M) values (S-protein: 6.4x10-10 ± 5.9x10-12; Sp5: 2.6x10-10 ± 4.7x10-12; Sp7: 4.1x10-10 ± 5.1x10-12) confirmed the equivalence of the three proteins in maintaining the wild-type conformational structure. After confirming the correct folding, the three viral proteins were characterized by an affinity assay toward the hACE2 receptor (Octet Red-Forte Bio).

***Manufacturing of wt Sp5 and Sp7 pseudo-viruses***

The recombinant Vesicular Stomatitis Virus (VSV) bearing SARS-CoV-2 Spike (S) protein in which the glycoprotein (G) gene has been replaced with the Green Fluorescent Protein (GFP) reporter gene (VSV-∆G-GFP-S) was generated in BHK-21 cells as previously described[^9^](#_ENREF_9). Similar protocol has been used for VSV-∆G-GFP-Sp5 and VSV-∆G-GFP-Sp7 mutants.

***Cells, treatments and infections***

EA.hy926 cells (ATCC) were cultured in complete medium: DMEM supplemented with penicillin-streptomycin, non-essential amino acids (NEAA, ThermoFisher Scientifics), tricine buffer (Sigma-Aldrich), HAT (Sigma-Aldrich), and 10% fetal bovine serum (FBS, HyClone, Thermo Scientific) on 0.2% gelatin-coated plates. Cells were seeded at a 150.000 cells/ml density in complete medium, and at confluence (after about 48 hours) were starved for 24 hours in fresh DMEM supplemented with 2% FBS. After starvation they were cultured for 48 hours in DMEM 0.5% FBS with TNFα (50 ng/ml, Peprotech), 17β-Estradiol (10 - 100 nM; Sigma-Aldrich), Raloxifene (2 µM; Sigma-Aldrich), Fulvestrant (100 nM; Sigma-Aldrich) and/or wild-type S-protein and, and the two Sp5/Sp7 mutants[^10^](#_ENREF_10). Trehalose (the protein stabilizer employed in wt-S, Sp5 and Sp7 preparation) was tested in preliminary experiments to assess possible toxic effects. In no case the addition of this compound to culture medium determined cell death (data not shown).

HuLEC-5a cells (ATCC) were cultured in complete medium: MCDB 131 (Gibco™) supplemented with 1% penicillin-streptomycin, 10 ng/mL epidermal growth factor (EGF, Peprotech), 1 µg/mL Hydrocortisone (Merck), 10 mM Glutamine, and 10% fetal bovine serum (EuroClone) on 12-well plates. Cells were seeded at a 150.000 cells/ml density, and at confluence (approximately after 48 hours) they were cultured in complete medium for 24 hours with TNFα (50 ng/ml, Peprotech), 17β-Estradiol (10 nM; Sigma-Aldrich), Raloxifene (2 µM; Sigma-Aldrich), Fulvestrant (100 nM; Sigma-Aldrich) and/or wild-type S protein and the two mutants Sp5 and Sp7. After 24 hours, cells were washed with PBS and lysed in TRIzol (Invitrogen).

For the infection with SARS-CoV-2, after starvation, the ECs were incubated with RAL (for 10 minutes) or FS (5 hours) at 37°C and after 1x PBS wash, exposed to the SARS-Cov-2 isolate (2019-nCoV/Italy-INMI1; EVAg, Ref-SKU: 008V-03893) at MOI 0.1 and MOI 1 for 1 hour at 37°C. Then, SARS-CoV-2 containing medium was removed and, after 2x wash with PBS, fresh culture medium containing RAL o FS was added for additional two days of culture. ECs supernatants were collected 48 h post infection to compare viral release among different experimental conditions by using the RT-qPCR assay (Simplexa™ COVID-19 Direct assay, Diasorin S.p.A) to detect and *ORF1ab* SARS-CoV-2 coding sequence[^11^](#_ENREF_11). Cells were washed with PBS and lysed in TRIzol (Invitrogen). Treatments were performed in six replicates for each experimental condition.

For the infection with the pseudo-viruses, EA.hy926 cells were seeded at a 50.000 cell/mL density in complete medium, and after 48 hours were starved for 24 hours in fresh DMEM supplemented with 2% FBS. After starvation, the cells were cultured for 48 hours in DMEM 1.8% FBS with SARS-CoV-2 Swt, SARS-CoV-2 Sp5 or SARS-CoV-2 Sp7 pseudoviruses at a multiplicity of infection (MOI) of 0.3. The effect of inhibitors was determined in cells pre-incubated with medium containing Raloxifene (2 µM, 10 minutes) or Fulvestrant (100 µM, 5 hours).

***Quantitative real-time polymerase chain reaction (qRT-PCR)***

For qRT-PCR experiments, cells were directly harvested in TRIzol Reagent (ThermoFisher Scientifics), while the electroporated adductor muscles of mice were collected in RNA*Later* (Sigma-Aldrich). For gene expression analysis in endothelial cells, 1 µg of RNA was reverse transcribed using iScript™ Advanced cDNA Synthesis Kit (Biorad). qRT-PCR was then carried out to assay TF (TF: forward 5’- CCCAAACCCGTCAATCAAGTC-3’, reverse 5’- CCAAGTACGTCTGCTTCACAT-3’) and 18S ribosomal RNA (18S: forward 5’-CGGCTACCACATCCAAGGAA-3’, reverse 5’- CCTGTATTGTTATTTTTCGTCACTACCT -3’) used as internal reference genes. Samples of cDNA (2.5 µL) were incubated with 25 µL of containing TF or 18S primers and fluorescent Luna® Universal qPCR Master Mix (New England Biolabs), and qRT-PCR was carried out in triplicate for each sample on the CFX Connect real-time System (Bio-Rad Laboratories). For RT-qPCR of Spike gene transfer, total RNA was extracted from murine adductor muscles following the conventional phenol-chloroform method. Superscript III (Thermo Fisher Scientific) was used for reverse transcription. RT-qPCR analyses were performed with Power SYBR Green PCR Master Mix (Applied Biosystems) in an ABI 7900 Fast thermal cycler to detect Spike (forward 5’- GTGTACTTCGCCTCTACCGA-3’, reverse 5’- GTGATAGTACACGCCCAGGA-3). The reported expression levels were calculated relative to GAPDH mRNA, used as an internal standard control (forward 5’- CTCCCACTCTTCCACCTTCG-3’, reverse 5’- GCCTCTCTTGCTCAGTGTCC-3).

***Procoagulant activity***

Samples were lysed with 15 mM n-Octyl-B-D-glucopyranoside lysis buffer at 37°C for 10 min, sonicated at 20 kHz for 20 seconds and diluted with 25 mM HEPES saline. The total protein concentrations were determined using the Bradford method. 60 µl of homogenate (0.07 mg/mL) were mixed with 60 µL citrated pooled human plasma and 60 µL CaCl_2_ (final concentration 25 mM), and procoagulant activity was quantified by a one-stage plasma recalcification time assay. Clotting times were expressed in relative U/µg protein based on a standard curve of serially diluted human thromboplastin preparation.

***TF detection***

ACTICHROME® TF activity assay was measured according to manual instructions (BioMedica Diagnostics). Briefly, cells were lysed in a recommended buffer (50mM Tris-HCl, 100mM NaCl, 0.1% Triton X-100, pH 7.4), sonicated for 10 seconds and incubated at 37°C for 30 minutes. TF activity was detected by two stage chromogenic assay interpolating the mean absorbance values of test samples, directly from the standard curve. TF cell supernatant was measured by IMUBIND® Tissue Factor ELISA kit according to manual instructions (BioMedica Diagnostics).

***Preparation of in vivo expression vector, in vivo gene transfer and pharmacological treatment***

In order to increase the level of transcription *in vivo*, an optimal translation initiation (Kozak) sequence was inserted upstream of the ATG. Two consecutive stop codons were inserted downstream of the codon sequence and upstream of bovine growth hormone (bGH) polyadenylation signal. The cDNA molecular design was based on a bioinformatic study and according to previous studies [^12^](#_ENREF_12)^,^[^13^](#_ENREF_13). Codon-optimized S-protein mutants were synthesized taking into account codon usage bias, GC content, CpG dinucleotides content, mRNA secondary structure, cryptic splice sites, premature PolyA sites, internal chi sites and ribosomal binding sites, negative CpG island, RNA instability motif (ARE), repeat sequences (direct repeat, reverse repeat and Dyad repeat) and restriction sites that may interfere with cloning. To increase the efficiency of translational termination, two consecutive stop codons were inserted at the end of cDNAs. The codon usage bias in human was upgraded to a CAI of at least 0.94. GC content and unfavorable peaks were optimized to prolong the half-life of the mRNA. The DNA for electorporation was formulated in Phosphate Buffered Saline (PBS). DNA-EP was performed by means of a Cliniporator Device EPS01 and using N-10-4B electrodes (IGEA, Italy) with the following electrical conditions in Electro-Gene-Transfer (EGT) modality: 8 pulses 20 msec each at 110V, 8Hz, 120msec interval. Mice with electroporation procedure only were used as control group. Groups of mice were treated with Raloxifene at the dose of 30mg/kg bodyweight by intraperitoneal administration (10µL/g bodyweight) of a 3 mg/mL Raloxifene solution dissolved in double distilled water containing 5% DMSO (Sigma-Aldrich), and 10% Cremophore EL (Sigma-Aldrich). Administration was performed 1 hour before and 24 hours after electroporation. Control animals received vehicle only (5% DMSO and 10% Cremophore EL in double distilled water).

***In vivo gene transfer and coagulation markers detection***

The *in vivo* study was carried out in accordance with UKCCCR guidelines for the welfare of animals as well as the European Directive 2010/63/EU. For *in vivo* expression and immunogenicity assessment, 6-8 weeks old C57BL/6 female mice (n=10-16/group, Envigo, USA) were injected intramuscularly (i.m.), particularly in the quadriceps, with either DNA plasmid or amplicon (dose ranging from 10 µg to 50 µg) and electrically stimulated as previously described [^13^](#_ENREF_13). Further details about gene transfer procedure, immunization against Spike, pharmacological treatment with Raloxifene, and detection of coagulation markers are provided in the supplementary material.

***Detection of coagulation markers and immunization***

After 24, 48 and 96 hours after electroporation, animals were anesthetized and blood collected by cardiac venipuncture into 3.8% sodium citrate. For plasma preparation citrated blood was centrifuged (within 30 minutes) at 3000 rpm for 20 min, and immediately stored in dry ice and then at -80 °C. For detection of the plasma clotting time, recalcification time was evaluated according to a method previously described [^14^](#_ENREF_14). Briefly, to 30 µL mouse platelet-poor plasma (PPP) were added 30 µL of HEPES saline buffer and 20 µL of citrated PPP obtained from pooled wild-type mouse. Samples, pre-wormed for 1 minute in the thermostatically-controlled water bath at 37°C, were mixed to 40 µL of CaCl_2_ 25mM and incubated at 37°C with shaking until clot formation. Clotting time was measured as the time plasma takes to form a stable clot, with the loss of any movement in response to the rotation and shaking applied by a blinded operator to the experimental groups. For *in vivo* detection of TF, D-dimer and VWF, commercially available kits were used to measure in citrated plasma the specific antigens, according to Manufacturer’s instruction (Cloud-Clone Corp).

For the ELIspot a commercial kit (Mabtech) was used. Briefly, splenocytes collected from mice inoculated with the DNA vectors 35 days earlier were plated at 250,000 and 500,000 in duplicate for each condition to be tested (DMSO, Pool S1, Pool S2, and Concanavaline A). The S1 and S2 peptides used as an overnight stimulus were used at a concentration of 1µg/ml; concanavaline A (C5275, Sigma) was used as a positive control at a concentration of 250 µg/ml. Results were reported as SCF (Spot Forming Cells)/10^6^ splenocytes. For sera titration an in-house ELISA assay was developed. Briefly, full length wild-type S-protein was coated on a 96well plate at a concentration of 1µg/ml in PBS in a final volume/well of 50 µl. The plate was incubated overnight at 4°C and after 5 washes with PBS1X-Tween 0,05% was blocked with 150μl of BSA3% in PBS1X-Tween 0,05% for 1hour at RT in agitation. Scalar dilutions of sera collected from transfected mice at day 35 (1:300; 1:900 1:2700 1:8100 1:24300 1:72900 1:218700) were then incubated overnight at 4°C. After 5 washes with PBS1X-Tween 0,05% an anti-mouse IgG (H + L)-HRP Conjugate (Biorad) diluted 1:2000 in PBS1X-Tween 0,05% was added for 1h at RT. Plate was developed with 50μl of Alkaline Phosphatase Yellow (pNPP, Sigma) for 30 minutes, and read at 405 nM in a Microplate reader (Tecan). Endpoint titer was calculated by plotting the log10 OD and the log10 sample dilution. A regression analysis of the linear part of the curve allowed calculation of the endpoint titer. An OD of 0.2 was used as a threshold.

***Pulmonary thromboembolism***

A disseminated thrombosis model was generated by a rapid intravenous injection into the tail veins of a mixture of collagen (550 μg/kg) and epinephrine (60 μg/kg) (Mascia Brunelli). Mice were observed for 10 min to assess the time of respiratory paralysis/distress that lasted at least 1 minutes or the cessation of breathing[^15^](#_ENREF_15). At the end of each experimental session, surviving animals were sacrificed by an overdose of anesthesia.

***Statistical analyses***

Statistical treatment of the data was performed using the Graph (Prism) software (version 9.0). Bar graphs were generated using the same program. The choice of statistical tests was done based on the possibility to perform paired/unpaired statistical tests using parametric/non-parametric tests following data normality checking with Kolmogorov-Smirnov test. The type of tests used as well as the level of significance is indicated in the figure legends and the figures, respectively. A *P* < 0.05 was considered an acceptable significance level to assess a statistically-validated difference among treatments/samples. The number of replicates included in in each analysis is indicted by the number of the dots overlapped to each bar in histogram plot.


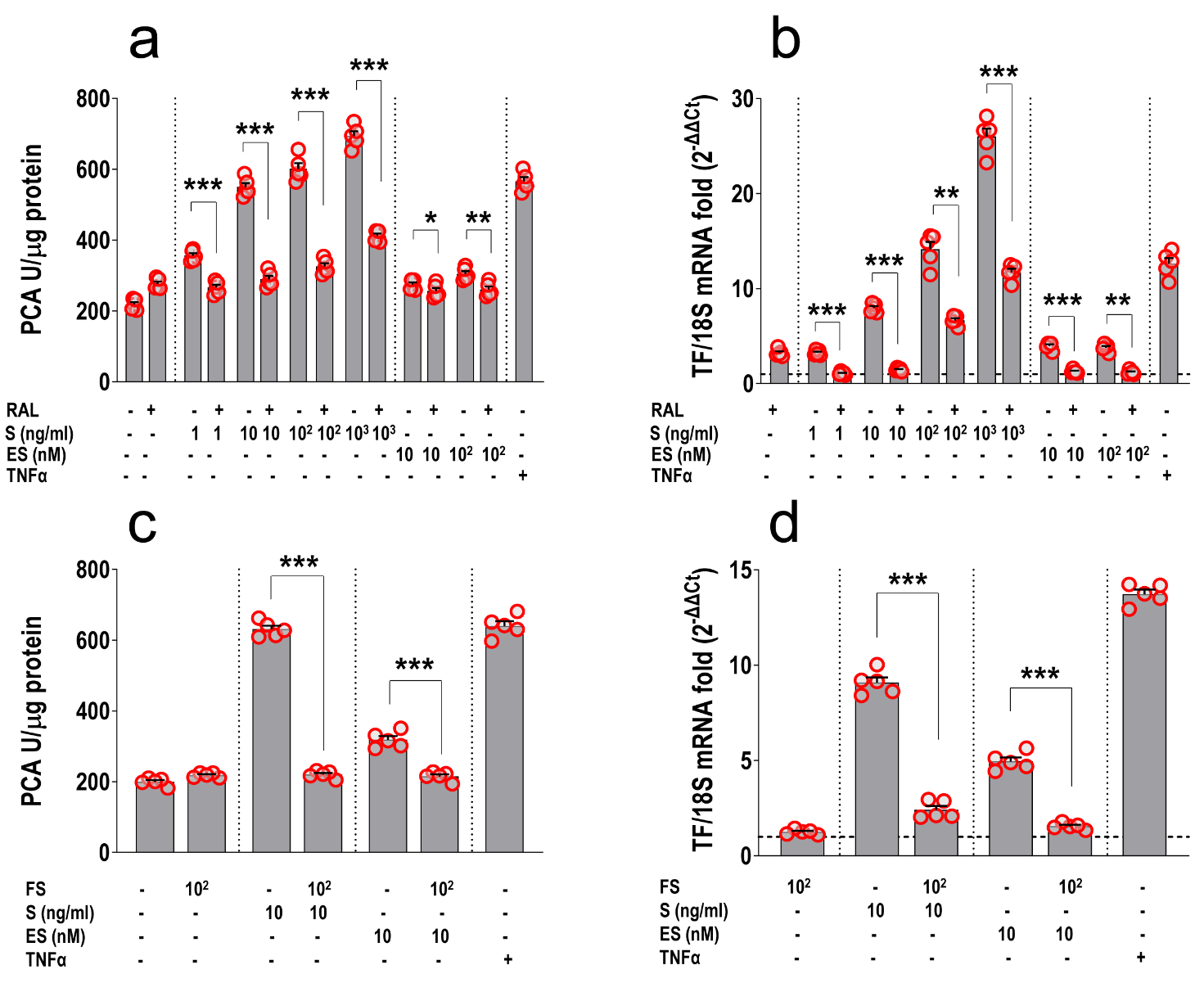


**Figure S1.** Pro-coagulant activity (PCA) and fold changes of Tissue Factor (*TF)* mRNA expression in ECs treated with, **a,** **b:** increasing doses of wild-type S-protein or 17β-Estradiol (ES) with or without Raloxifene (RAL), and Tumor Necrosis Factor alpha (TNFα). **c, d:** with S-protein (10 ng/ml) and ES ± Fulvestrant (FS). The S-protein concentrations used in these experiments were in line with the reported level of circulating Spike in COVID-19 patients[^16^](#_ENREF_16), and well above the maximal values measured after inoculating the currently available mRNA vaccines (≤0.1ng/mL). Dotted line in **b, d** indicate the level of *TF* mRNA expression in the controls, equalized to 1 to make possible representation of fold chances *vs.* controls. ***P* < 0.01 and ****P* < 0.0001, as calculated by paired Student’s t-test in each of the treated *vs.* inhibitor-treated cells. The number of experimental replicates is indicated by the individual circles overlapped to graphs.


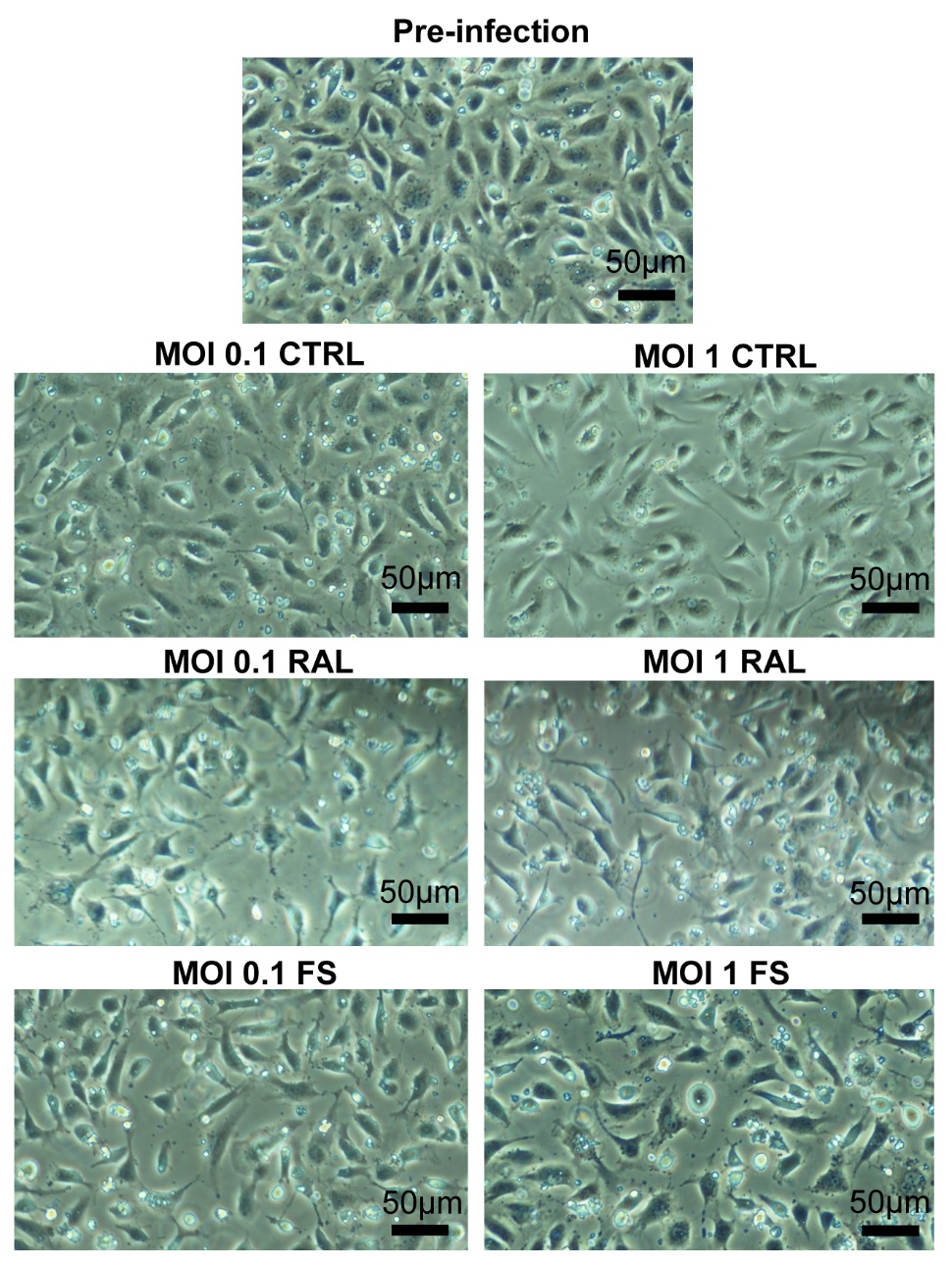


**Figure S2. Effects of the two ERα inhibitors** **Raloxifene and Fulvestrant on SARS-CoV-2 infected ECs.** Representative optical images of control (Pre-infection, top single panel) and SARS-CoV-2 infected (MOI 0.1 CTRL and MOI 1 CTRL) ECs treated with Raloxifene (RAL) or Fulvestrant (FS) taken 48h post infection. A mild cytopathic effect, characterized by cell rounding and wrinkling, was observed mainly with RAL compared to FS, consistently with viral shedding measured in supernatants (**Figure 1b**). Magnification 10x.

**
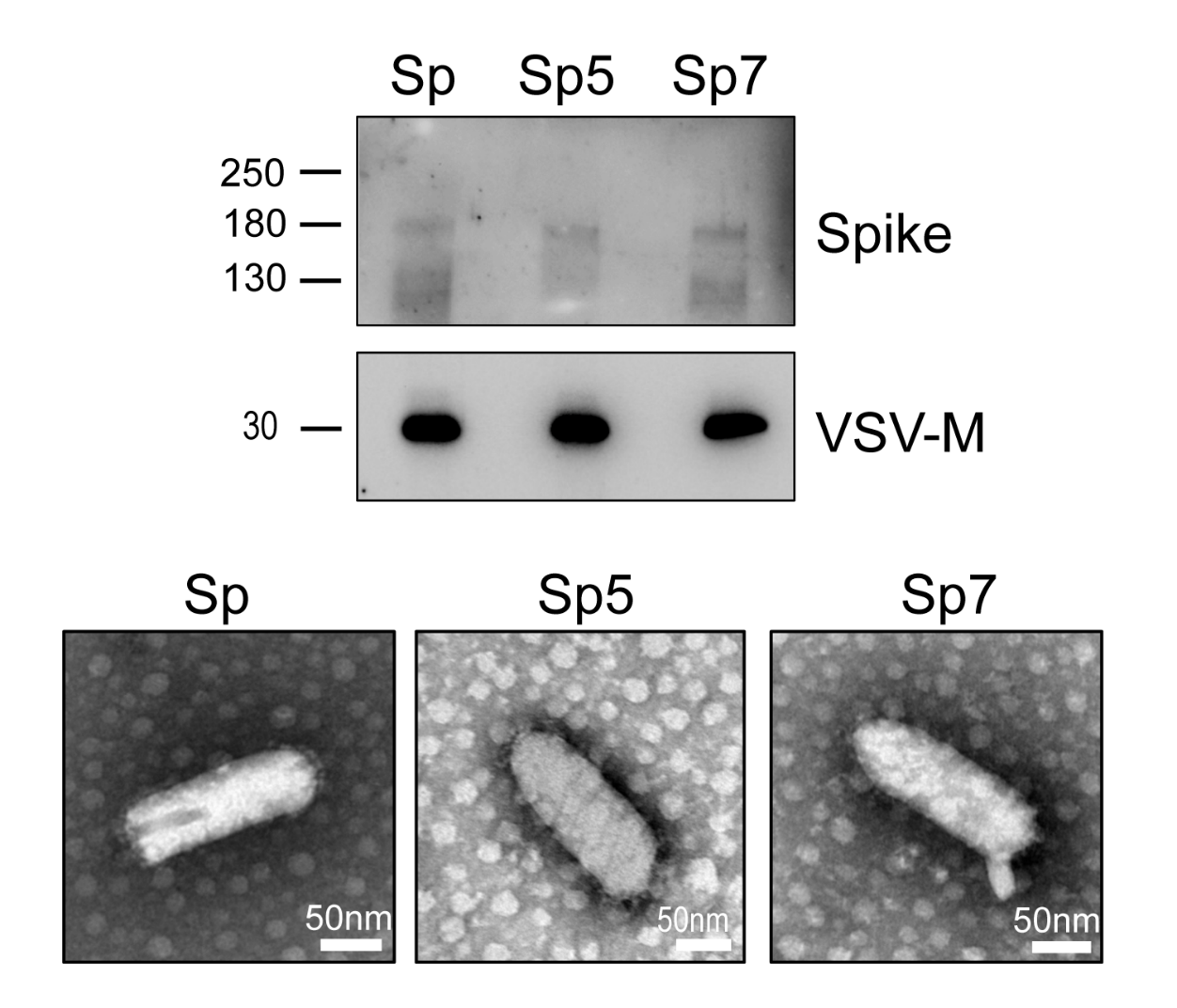
**

**Figure S3.** Characterization of VSV-ΔG-GFP-Sp wt (Sp), VSV-ΔG-GFP-Sp5 (Sp5) and VSV-ΔG-GFP-Sp7 (Sp7) pseudoviruses. Upper: Western blot analysis of Spike protein incorporation on VSV viral membranes using an anti-Spike antibody for Sp, Sp5 and Sp7 pseudoviruses. VSV Matrix (VSV-M) proteins used as a loading control for viral particles. Lower: representative images of Negative Staining electron microscopy to visualize the Spike proteins in Sp, Sp5 and Sp7 pseudoviruses. Scale bars, 50 nm.


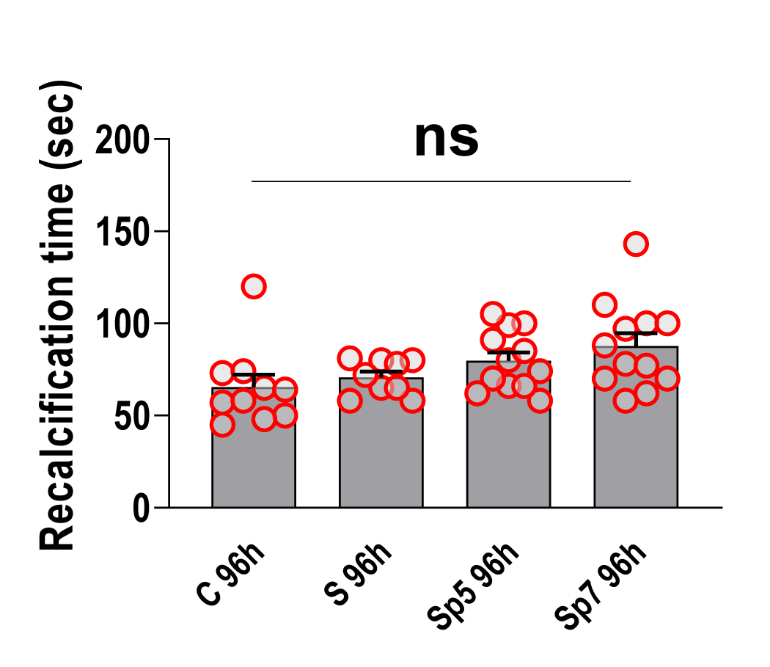


**Figure S4.** Recalcification time of plasma obtained by mice inoculated with the wt S-protein, the Sp5 and Sp7 mutants at 96 hours post transfer. Analysis by one-way ANOVA with Tukey post-hoc did not reveal statistically significant differences between groups (ns).

**Supplementary references**

1 Borocci, S. *et al.* Altered Local Interactions and Long-Range Communications in UK Variant (B.1.1.7) Spike Glycoprotein. *Int J Mol Sci* **22**, 5464, doi:10.3390/ijms22115464 (2021).

2 Delfosse, V. *et al.* Structural and mechanistic insights into bisphenols action provide guidelines for risk assessment and discovery of bisphenol A substitutes. *Proc Natl Acad Sci U S A* **109**, 14930-14935, doi:10.1073/pnas.1203574109 (2012).

3 Tagliamonte, M. S. *et al.* Multiple Recombination Events and Strong Purifying Selection at the Origin of SARS-CoV-2 Spike Glycoprotein Increased Correlated Dynamic Movements. *International Journal of Molecular Sciences* **22**, 80, doi:10.3390/ijms22010080 (2021).

4 Maier, J. A. *et al.* ff14SB: Improving the Accuracy of Protein Side Chain and Backbone Parameters from ff99SB. *Journal of chemical theory and computation* **11**, 3696-3713, doi:10.1021/acs.jctc.5b00255 (2015).

5 Kirschner, K. N. *et al.* GLYCAM06: a generalizable biomolecular force field. Carbohydrates. *J Comput Chem* **29**, 622-655, doi:10.1002/jcc.20820 (2008).

6 Mocklinghoff, S. *et al.* Synthesis and crystal structure of a phosphorylated estrogen receptor ligand binding domain. *Chembiochem* **11**, 2251-2254, doi:10.1002/cbic.201000532 (2010).

7 Yan, Y., Tao, H., He, J. & Huang, S. Y. The HDOCK server for integrated protein-protein docking. *Nat Protoc* **15**, 1829-1852, doi:10.1038/s41596-020-0312-x (2020).

8 Olsson, C., Jansson, H. & Swenson, J. The Role of Trehalose for the Stabilization of Proteins. *The journal of physical chemistry. B* **120**, 4723-4731, doi:10.1021/acs.jpcb.6b02517 (2016).

9 Condor Capcha, J. M. *et al.* Generation of SARS-CoV-2 Spike Pseudotyped Virus for Viral Entry and Neutralization Assays: A 1-Week Protocol. *Front Cardiovasc Med* **7**, 618651, doi:10.3389/fcvm.2020.618651 (2020).

10 Patel, H. K. & Bihani, T. Selective estrogen receptor modulators (SERMs) and selective estrogen receptor degraders (SERDs) in cancer treatment. *Pharmacol Ther* **186**, 1-24, doi:10.1016/j.pharmthera.2017.12.012 (2018).

11 Amendola, A. *et al.* Human cardiosphere-derived stromal cells exposed to SARS-CoV-2 evolve into hyper-inflammatory/pro-fibrotic phenotype and produce infective viral particles depending on the levels of ACE2 receptor expression. *Cardiovasc Res* **117**, 1557-1566, doi:10.1093/cvr/cvab082 (2021).

12 Watanabe, Y. *et al.* Native-like SARS-CoV-2 Spike Glycoprotein Expressed by ChAdOx1 nCoV-19/AZD1222 Vaccine. *ACS Cent Sci* **7**, 594-602, doi:10.1021/acscentsci.1c00080 (2021).

13 Conforti, A. *et al.* COVID-eVax, an electroporated DNA vaccine candidate encoding the SARS-CoV-2 RBD, elicits protective responses in animal models. *Mol Ther* **30**, 311-326, doi:10.1016/j.ymthe.2021.09.011 (2022).

14 Cao, L. *et al.* Plasma-deposited tetraglyme surfaces greatly reduce total blood protein adsorption, contact activation, platelet adhesion, platelet procoagulant activity, and in vitro thrombus deposition. *J Biomed Mater Res A* **81**, 827-837, doi:10.1002/jbm.a.31091 (2007).

15 Barbieri, S. S. *et al.* Abnormal megakaryopoiesis and platelet function in cyclooxygenase-2-deficient mice. *Thrombosis and haemostasis* **114**, 1218-1229, doi:10.1160/TH14-10-0872 (2015).

16 Avolio, E. *et al.* The SARS-CoV-2 Spike protein disrupts human cardiac pericytes function through CD147 receptor-mediated signalling: a potential non-infective mechanism of COVID-19 microvascular disease. *Clin Sci (Lond)* **135**, 2667-2689, doi:10.1042/CS20210735 (2021).
